# Supplementary material for: Energy-efficient high-fidelity image reconstruction with memristor arrays for medical diagnosis
Source: Nat Commun. 2023 Apr 20;14:2276. doi: 10.1038/s41467-023-38021-7 (PMC10119144; doi:10.1038/s41467-023-38021-7)
Supplement: Supplementary file 1 — Supplementary Information [file 41467_2023_38021_MOESM1_ESM.pdf]

Supplementary Information

Energy-efficient high-fidelity image reconstruction with memristor arrays for medical diagnosis

Han Zhao[1,†], Zhengwu Liu[1,†], Jianshi Tang[1,2*], Bin Gao[1,2], Qi Qin[1], Jiaming Li[1], Ying Zhou[1], Peng Yao[1], Yue Xi[1], Yudeng Lin[1], He Qian[1,2], Huaqiang Wu[1,2]

[1]School of Integrated Circuits, Beijing National Research Center for Information Science and Technology (BNRist), Tsinghua University, Beijing, 100084, China.

[2]Beijing Innovation Center for Future Chips (ICFC), Tsinghua University, Beijing, 100084, China.

[†]These authors contributed equally: Han Zhao, Zhengwu Liu.

[*]E-mail: jtang@tsinghua.edu.cn

This **Supplementary Information** contains:

Figure S1. Illustrations of QAM and QM.

Figure S2. Illustration of memristor read noise.

Figure S3. The impact of cumulative errors on MIR.

Figure S4. Implementation of DFTs with different numbers of point by software and MIR.

Figure S5. Implementation of 2D DFT.

Figure S6. Impact of memristor stuck-at faults.

Table S1. Detailed benchmarks of MIR for MRI and CT image reconstruction tasks.

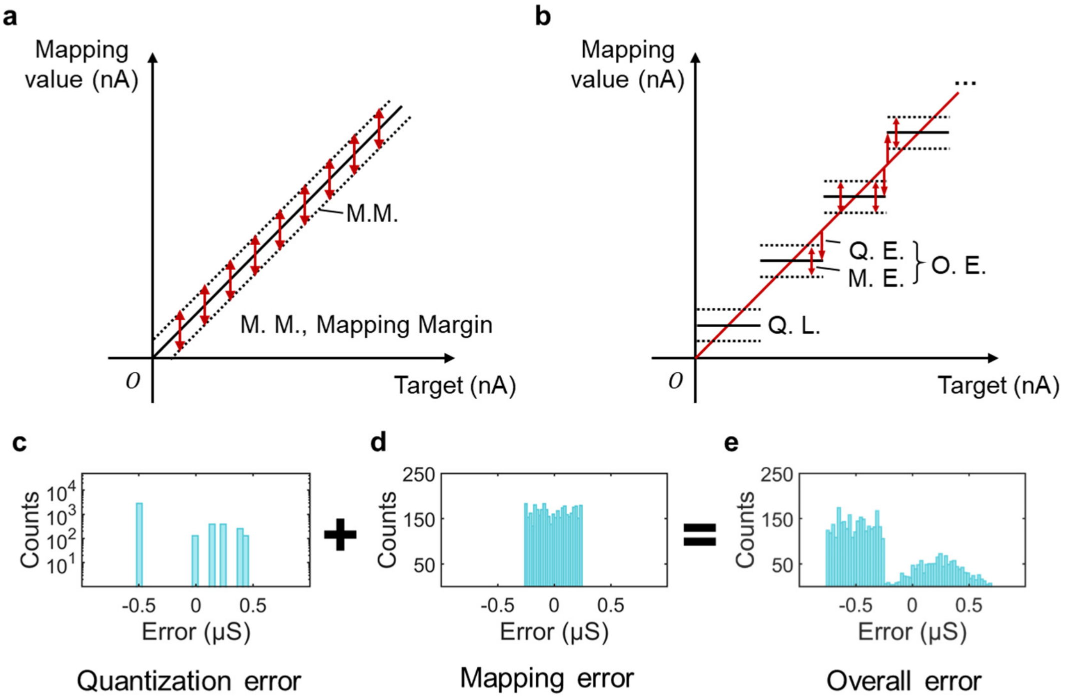

Supplementary Figure 1| Illustrations of QAM and QM. a, QAM strategy. A high mapping precision can be achieved because the mapping error is only introduced by the deliberately defined mapping margin (M. M.). **b**, QM strategy. It results in much lower mapping precision because the overall error (O. E.) now consists of two parts, the quantization error (Q. E.) and the mapping error (M. E.) introduced by the pre-defined mapping margin. Q. L., quantization level. **c-e**, Illustration of the overall mapping error of QM (**e**), which combines the quantization error (**c**) and mapping error (**d**, with a pre-defined mapping margin of 0.25 μS) together.

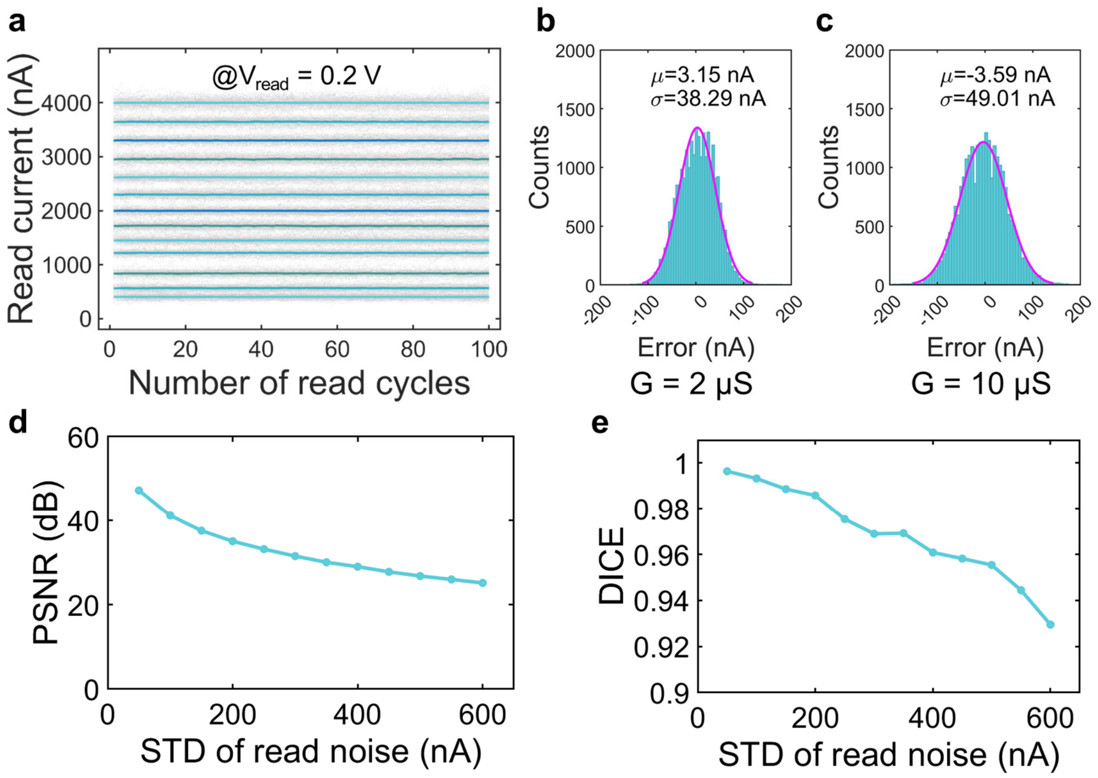

Supplementary Figure 2| Illustration of memristor read noise. a, Illustration of read noise of several representative conductance levels (256 devices are experimentally measured for each level). The gray lines represent the read current of each individual device and the colored lines represent the average values of each level. Here, the read voltage is 0.2 V. **b, c,** The error distribution of two conductance levels (2 μS and 10 μS). Both follow a Gaussian distribution. **d, e,** Simulation results (**d**, PSNR; **e**, DICE) of different levels of read noise. It is suggested that the read noise would not seriously degrade the image quality in this work because the typical value of the standard deviation (STD) of read noise is 50-100nA for our memristor devices.

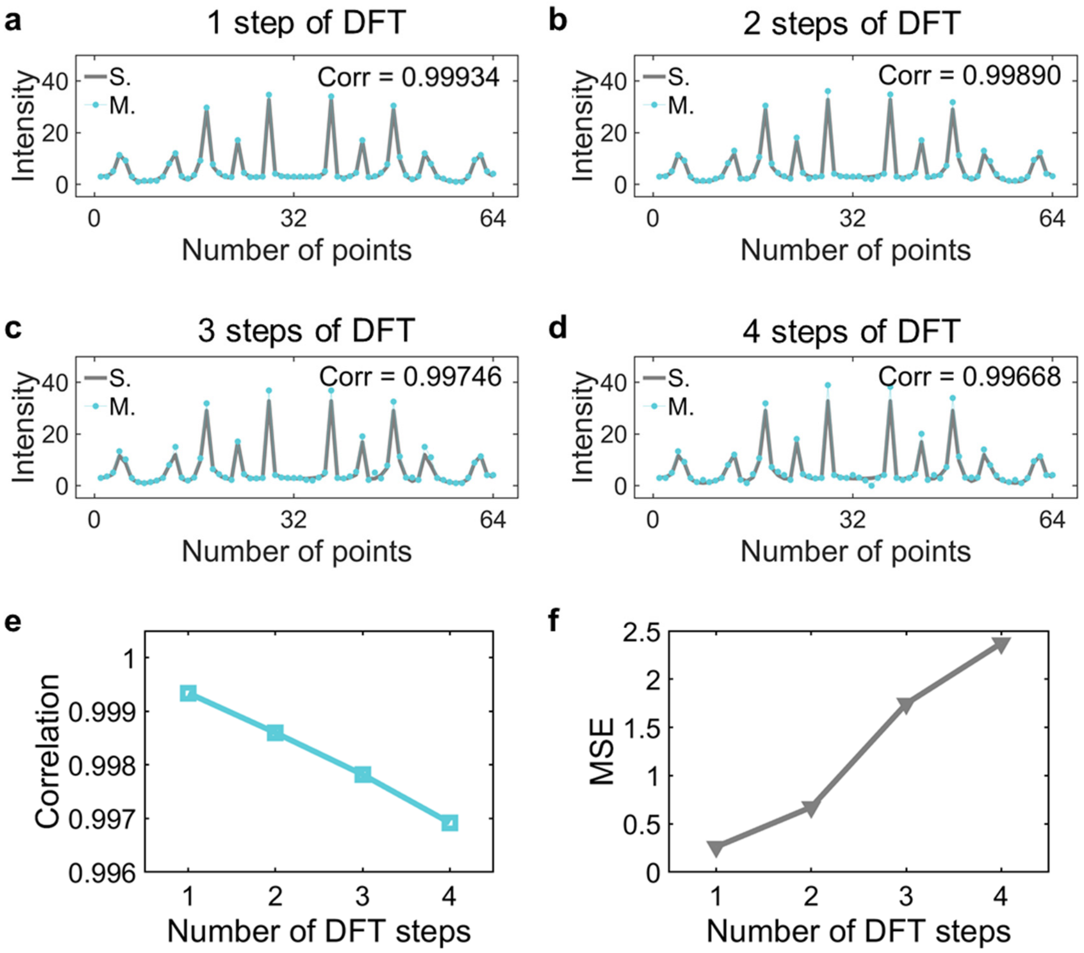

Supplementary Figure 3| The impact of cumulative errors on MIR. a-d, The computing results of 1, 2, 3 and 4 steps of DFT. Here, after a step of DFT is implemented on MIR, the frequency-domain signal is transformed back to time/spatial domain by lossless software IDFT, and the next step of DFT can be again performed by MIR. Corr, correlation coefficient. **e, f,** the correlation coefficient and mean squared error (MSE) of frequency-domain signal after a series of DFT steps.

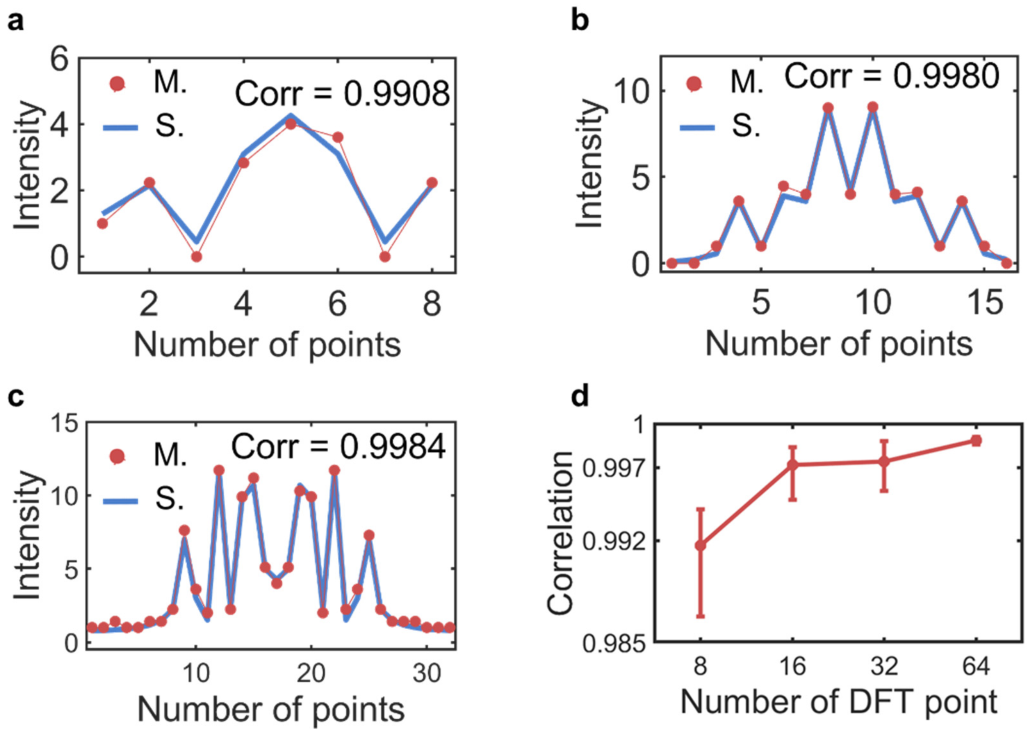

Supplementary Figure 4| Implementation of DFTs with different numbers of point by software (S.) and MIR (M.). a, 8-point DFT. Corr, correlation coefficient. **b**, 16-point DFT. **c**, 32-point DFT. **d**, Correlation coefficient of the computing results of MIR and software DFTs with different number of points. This experiment is repeated for 500 times to obtain statistical results. The circle represents the average correlation value of each scenario, while the error bar indicates the maximum and minimum values.

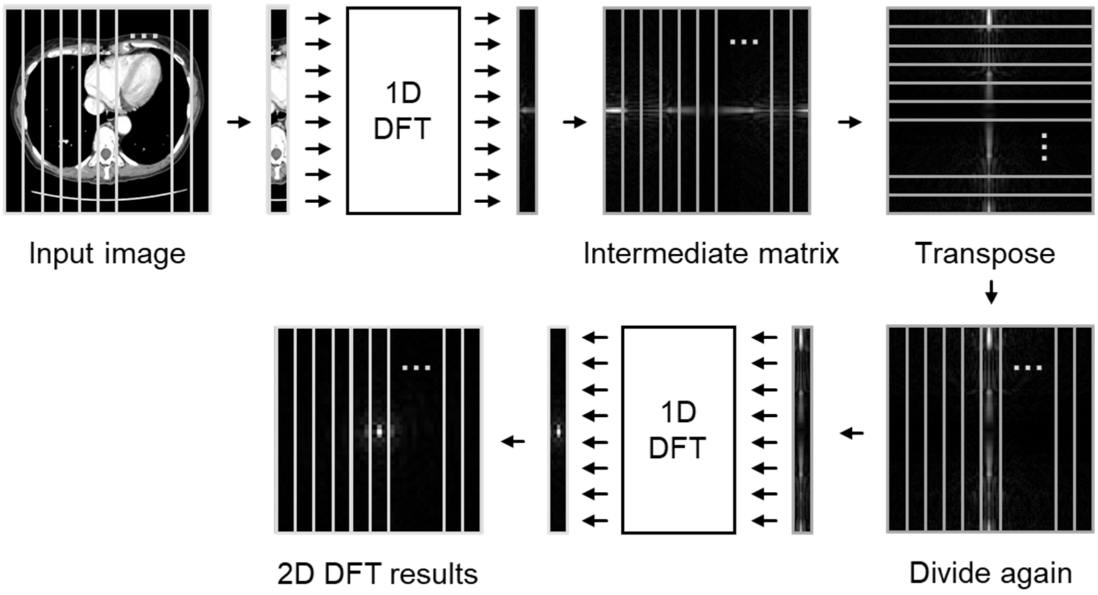

Supplementary Figure 5| Implementation of 2D DFT. 2D DFT can be performed by implementing several 1D DFTs. The input image is first divided into column vectors and 1D DFT is implemented for each vector. The computing results of 1D DFT are composed into an intermediate matrix. After transpose, the intermediate matrix is again divided into column vectors and a second 1D DFT is performed, whose output represents the results of 2D DFT.

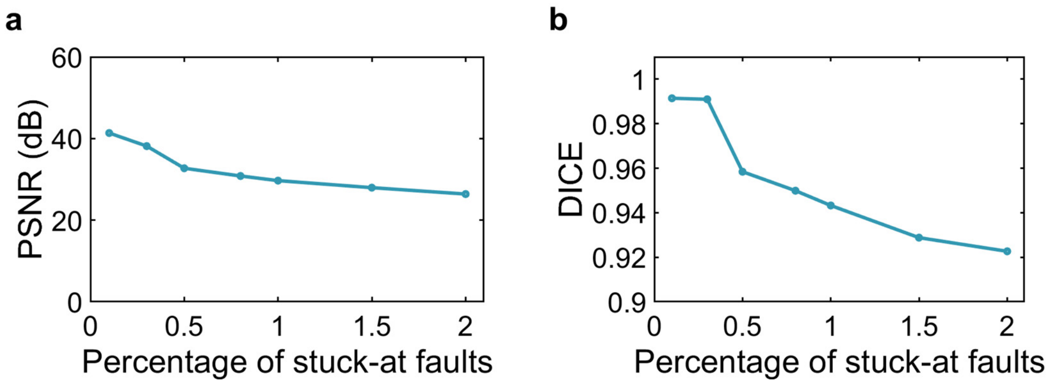

Supplementary Figure 6| Impact of memristor stuck-at faults. Simulation results (**a**, PSNR; **b**, DICE) with different percentages of stuck-at faults, indicating the importance of achieving a high yield. A large degradation of PSNR and DICE occurs at the range of 0.1%-0.5%. However, after extensive efforts in the process development, the typical yield of our memristor arrays is ~99.99% (the percentage of stuck-at faults is ~0.01%). Hence, the impact of stuck-at faults can be mostly ignored in this work.

a

MIR for MRI reconstruction task			
**Module**	**Number**	**Area/mm^2**	**Energy/nJ**
BL driver	1280	0.26	6.01
WL driver	1280	0.07	1.34
Memristor array	800K	0.22	36.04
Sample & hold	640	4.16	26.75
ADC	320		
Shift adder	320	0.44	4.68
**Sum**	**-**	**5.15**	**74.83**

b

MIR for CT reconstruction task			
**Module**	**Number**	**Area/mm^2**	**Energy/nJ**
BL driver	3072	1.25	32.20
WL driver	3072	0.18	1.49
Memristor array	4.5M	1.28	207.62
Sample & hold	1536	9.98	64.20
ADC	768		
Shift adder	768	1.05	11.23
**Sum**	**-**	**13.73**	**316.76**

Supplementary Table 1| Detailed benchmarks of MIR for MRI and CT image reconstruction tasks. a, Area and energy consumption breakdown of one 320-point IDFT with MIR for the MRI image reconstruction task. BL, bit line. WL, word line. **b,** Area and energy consumption breakdown of one 768-point DFT/IDFT with MIR for the CT image reconstruction task.
